# Pleurodesis and Immunotherapy in NSCLC; Medical Thoracoscopy or VATS?

**DOI:** 10.7150/jca.40004

**Published:** 2020-01-14

**Authors:** Paul Zarogoulidis, Haidong Huang, Meng Yang, Jun Zhou, Yang Jiao, Qin Wang, Dimitris Petridis, Konstantinos Sapalidis, Chrysanthi Sardeli, Parthenopi Konsta, Charilaos Koulouris, Nikolaos Michalopoulos, Dimitrios Giannakidis, Nikolaos Barbetakis, Athanasios Katsaounis, Wolfgang Hohenforst-Schmidt, Aikaterini Amaniti, Savvas Petanidis, Kosmas Tsakiridis, Nikolaos Courcoutsakis, Alexandru Marian Goganau, Anastasios Vagionas, Konstantinos Romanidis, Panagoula Oikonomou, Michael Karanikas, Iason Nikolaos Katsios, Isaak Kesisoglou, Christoforos Kosmidis

**Affiliations:** 1 3rd Department of Surgery, “AHEPA” University Hospital, Aristotle University of Thessaloniki, Medical School, Thessaloniki, Greece; 2 Department of Respiratory & Critical Care Medicine, Changhai Hospital, the Second Military Medical University, Shanghai, P. R. China.; 3 Department of Respiratory, Changzhou 1st People's Hospital, The Third Affiliated Hospital of Soochow University, Changzhou, Jiangsu Province, P.R. China.; 4 Department of Food Technology, School of Food Technology and Nutrition, Alexander Technological Educational Institute, Thessaloniki, Greece.; 5 Department of Pharmacology & Clinical Pharmacology, School of Medicine, Faculty of Health Sciences, Aristotle University of Thessaloniki, Thessaloniki, Greece; 6 Intensive Care Unit, “AHEPA” University Hospital, Aristotle University of Thessaloniki, Medical School, Thessaloniki, Greece; 7 Thoracic Surgery Department, “Theageneio” Cancer Hospital, Thessaloniki, Greece; 8 Sana Clinic Group Franken, Department of Cardiology / Pulmonology / Intensive Care / Nephrology, “Hof” Clinics, University of Erlangen, Hof, Germany; 9 Anesthisiology Department, “AHEPA” University Hospital, Aristotle University of Thessaloniki, Medical School, Thessaloniki, Greece; 10 Department of Pulmonology, I.M. Sechenov First Moscow State Medical University, Moscow, 119992, Russian Federation.; 11 Thoracic Surgery Department, Interbalkan ``European`` Medical Center, Thessaloniki, Greece; 12 Radiology Department, University General Hospital of Alexandroupolis, Democritus University of Thrace, Alexandroupolis, Greece; 13 General Surgery Clinic 1, University of Medicine and Pharmacy of Craiova, Craiova County Emergency Hospital, Craiova, Romania; 14 Oncology Department, General Hospital of Kavala, Kavala, Greece; 15 Second Department of Surgery, University Hospital of Alexandroupolis, Medical School, Democritus University of Thrace, Alexandroupolis, Greece; 16 Department of Surgery, Democritus University of Thrace, Dragana, Alexandroupolis, Greece

**Keywords:** Medical Thoracoscopy, Pleurodesis, NSCLC, VATS

## Abstract

**Introduction**: Immunotherapy is a treatment option for non-small cell lung cancer advanced disease. However; immunotherapy in several patients induces orogonitis and effusion in different cavities. It is up to the treating physician to understand whether there is effusion due to adverse effect or disease progression. Pleurodesis in both cases might be a solution for recurrent pleura effusion.

**Patients and Methods**: Three hundred and thirty seven non-small cell lung cancer patients with adenocarcinoma and pleura effusion during first line immunotherapy treatment underwent medical thoracoscopy or Video assisted thoracic surgery (VATS) for pleurodesis with talk poudrage. Uniportal medical thoracoscopy was performed under general with dual channel endotracheal tube in one hundred and eleven patients. Video assisted thoracic surgery was performed in one hundred and eighty seven patients and conversion from medical to VATS procedure was done to thirty nine patients. All patients had stage IV disease with pleura involvement and were under first line pembrolizumab treatment with 200mg (PD-L1 ≥ 50%).

**Results**: The quantitative parameters of the study (expression, PY and cycle) were converted to an ordinal scale to facilitate the performance of statistical analysis. All parameters were examined as dependent against the parameter technique acting as independent to detect potential relationships. The results of multi Y versus X relationship revealed no statistically significant effect (p>0.05) of the three levels of technique against any response considered. Thus we can infer, quite safely, that the innovative operation (level 0) does not differ from the other two conventional methods (levels 2 and 3) through all parameters entered in the model. There was no significant difference between the different pleurodesis techniques.

**Discussion**: Immunotherapy is known to induce in a number of patients pleura effusion and pericarditis. However; pleurodesis is efficient when the appropriate method is performed to every patient. Careful assessment in a case by case manner has to be performed for each patient before any procedure is performed.

## Introduction

Non-small cell lung cancer (NSCLC) is still diagnosed at advanced stage disease. We have several treatment modalities for advanced stage disease with immunotherapy being the latest addition.^1^ Proper screening and lung cancer prevention is the next major issue to properly address for every smoker.^2^ Currently we have novel diagnostic tools and methods, such as; radial endobronchial ultrasound, convex-probe endobronchial ultrasound, electromagnetic navigation, cone beam CT guided bronchoscopy, transthoracic ultrasound biopsy, CT guided biopsy and medical thoracoscopy.^3^-^10^ Immunotherapy is administered as first line treatment with the drug pembrolizumab if the programmed death-ligand 1 (PD-L1) is expressed ≥50% in NSCLC with metastatic disease indifferent of adenocarcinoma or squamous.^11^ It is known that immunotherapy treatment has several adverse effects such as; pleura effusion, pericarditis, skin reactions, flu-like symptoms, pneumonitis, edema, hypothyroidism and sinus congestion. Medical thoracoscopy is both a diagnostic and therapeutic technique when pleurodesis is performed for repeated pleura effusion. It can be performed by either pulmonary physicians or thoracic surgeons. All cases cannot be performed by pulmonary physicians, in the case for example when we have advanced empyema or extensive fibrinous tissue, for all those cases that a second or a third portal is necessary in order to properly perform diagnosis of pleura effusion due to malignancy or perform pleurodesis. Pulmonary physicians are educated to a level where they can perform uniportal thoracoscopy under local sedation or in some cases under general anesthesia. In the case where upgraded techniques and instruments that thoracic surgeons use then thoracoscopy is performed by thoracic surgeons.^12^,^13^ In the current study we performed pleurodesis for NSCLC adenocarcinoma patients with advanced metastatic disease and under pembrolizumab immunotherapy and evaluated whether immunotherapy had a negative effect on the procedure.

## Patients and Methods

### Patients

Three hundred and thirty seven patients were included in this multicenter retrospective study diagnosed with non-small cell lung cancer adenocarcinoma. All patients were stage IV with metastatic disease and had programmed death-ligand 1 (PD-L1) ≥ 50% and were under pembrolizumab treatment with 200mg.^11^ During their treatment they presented recurrent pleura effusion and pleurodesis was decided. Recurrent was defined according to current guidelines.^14^ The pleural effusion was attributed either to disease progression or as an adverse effect due to immunotherapy treatment.

### Methods

**Figure 1:**
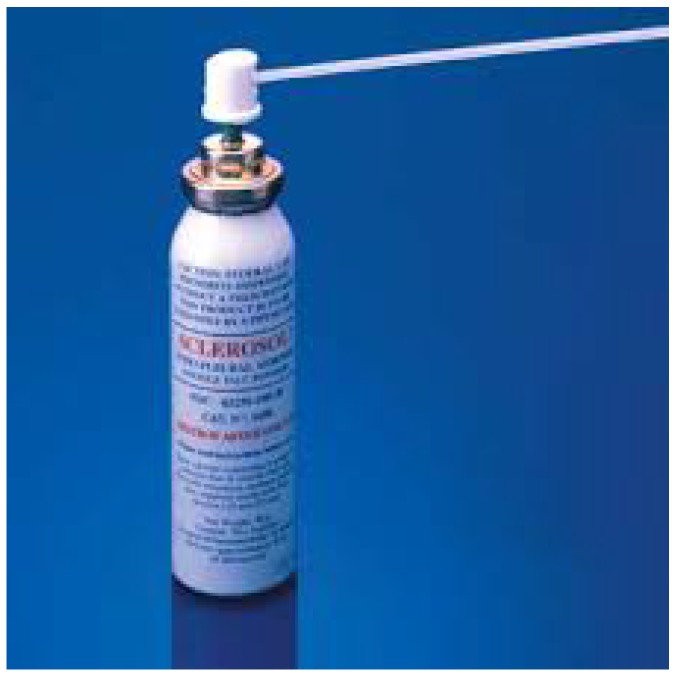
Aerosol talk poudrage

Based on every center`s experience and thoracic surgeon availability medical thoracoscopy was performed with dual channel tracheal tube and under general anesthesia in one hundred and eleven patients. One hundred and eighty seven patients underwent video assisted thoracic surgery-(VATS) again with dual channel tracheal tube and general anesthesia. In each patient talk poudrage in an aerosol form was used (Figure 1).

**Figure 2:**
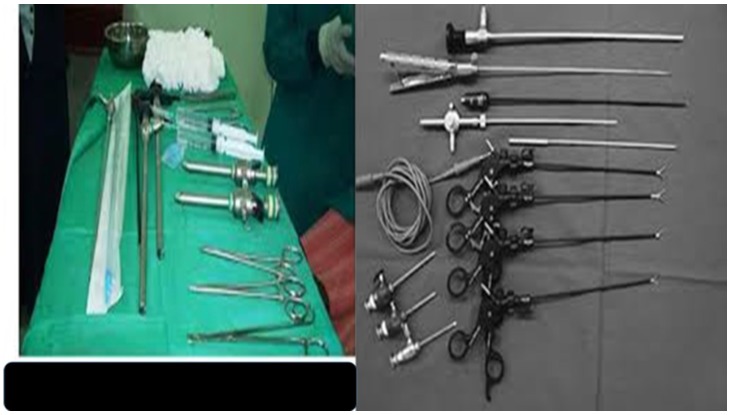
Left figure, medical thoracoscopy tools usually used by pulmonary physicians, right; tools usually used by thoracic surgeons

We chose the patients that were included in the study in a manner were exactly the same talk agent was used. Moreover; all patients underwent CT-scan of the thorax at least two days before the procedure and transthoracic ultrasound the same day with the procedure. All patients had positive pleura effusion with adenocarcinoma. We wanted to check whether there was empyema or fibrotic tissue between the visceral and parietal pleura. All patients were cleared from a cardiology perspective as fit to undergo any procedure. In this manner we wanted to predict whether a medical procedure would be upgraded to VATS and take any necessary precautions. Indeed, in thirty nine patients the medical thoracoscopy was upgraded to VATS due to technical reasons (Figure 2).

**Figure 3:**
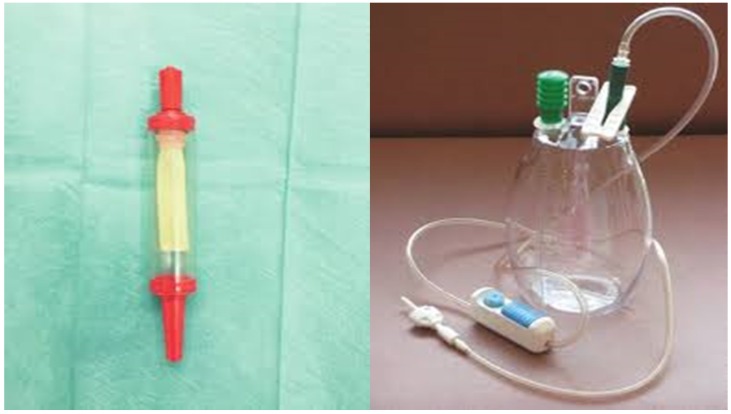
Left; Heimlich valve, right; Redon draining system

**Figure 4:**
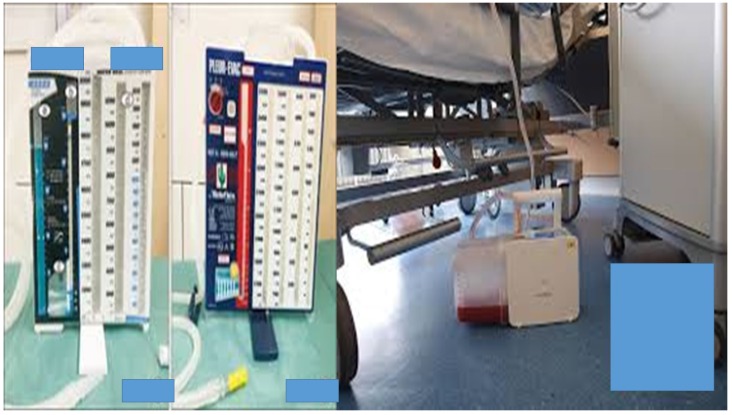
Left; simple water draining system for air and fluids of thoracic cavity, right; automatic draining system for air and fluids of thoracic cavity

In the case that pelurodesis was not achieved by any method then a pleurocatheter with a Heimlich valve was placed with a ``REDON`` draining system or a chest tube connected to a simple or electronic draining system (Figure 3,4).

## Results

The quantitative parameters of the study (expression, PY and cycle) were converted to an ordinal scale to facilitate the performance of statistical analysis. All parameters were examined as dependent against the parameter technique acting as independent to detect potential relationships.

The results of multi Y versus X relationship revealed no statistically significant effect (p>0.05) of the three levels of technique against any response considered (Table 1).

Thus we can infer, quite safely, that the innovative operation (level 0) does not differ from the other two conventional methods (levels 2 and 3) through all parameters entered in the model. Primarily, the operations corresponded proportionally between the success/failure of the outcome/result (Table. 2), producing equal operative performance, that is 87% versus 13% approximately.

Expression did not change significantly among the three methods (Table 3) and so did the number of metastasis (Table 4).

The pack-year was divided into three parts (0=non-smokers, 1= 1-160 moderately smokers and 2=>160 heavily smokers) and their frequency distribution was arranged according to the technique levels (Table 5).

Six adverse effects were recorded with no particular response of any against the surgery methods (Table 6).

The number of cycles was divided into three parts (1=1-8, 2=9-12 and 3=>12 cycles) and were tabulated with the technique (Table 7). Higher cycles indicate lower participation in the records across the operative methods.

Finally, Staging (Table 8) and noxious pleura (Table 9) were not affected by surgery, the latter indicating a ratio of 3/1 (0 versus 1) across the operative levels.

## Discussion

We chose to include in our study only adenocarcinoma as it has been observed from previous studies that this specific NSCLC type can easily infiltrate the pleura in the course of the disease and it usually induces pleura effusion in the course of the disease.^15^ Moreover; it has a different biological behavior from other NSCLC subtypes. We wanted primarily to investigate if immunotherapy is a major factor for pleurodesis failure and the efficiency of medical thoracoscopy performed by pulmonary physicians and VATS performed by thoracic surgeons. Medical thoracoscopy can be performed with local sedation in some patients for pleurodesis, however; if the candidate is not properly selected then the procedure is not successful.^16^ The centers that collaborated for this study performed the ``medical thoracoscopy`` under general anesthesia with dual channel tracheal tube in order to have a better visualization of the pleura and make the application of the aerosol talk more efficient.

Major limitation of the study was that we did have information from all patients regarding the subpopulations of the pleura fluid such as; eosinophils, LDH, proteins. We believe that the small rate of the patients that had their procedure converted from medical thoracoscopy to VATS was low due to the fact that the patients were carefully selected based on their performance status, CT findings (fibrotic tissue, atelectasis), respiratory status and heart condition. There was no statistical difference between the two methods in regards to success rate and none of the factors included in the study did not appear to affect the outcome. Also, there was no difference of pleurodesis success rate between different PD-L1 expression. In all patients the pleural effusion was malignant and whether there was also additionally orogonitis, it did not affect in any way the outcome of pleurodesis. In a future study we will investigate different types of NSCLC and PD-L1 expression with different immunotherapy treatments and dosages, in order to investigate if there is any association.

## Figures and Tables

**Table 1:**
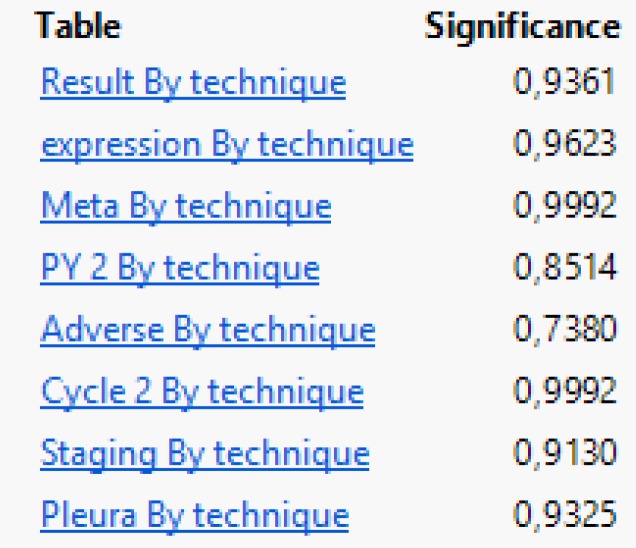
Exact probability values of the effect of technique against each parameter under study.

**Table 2:**
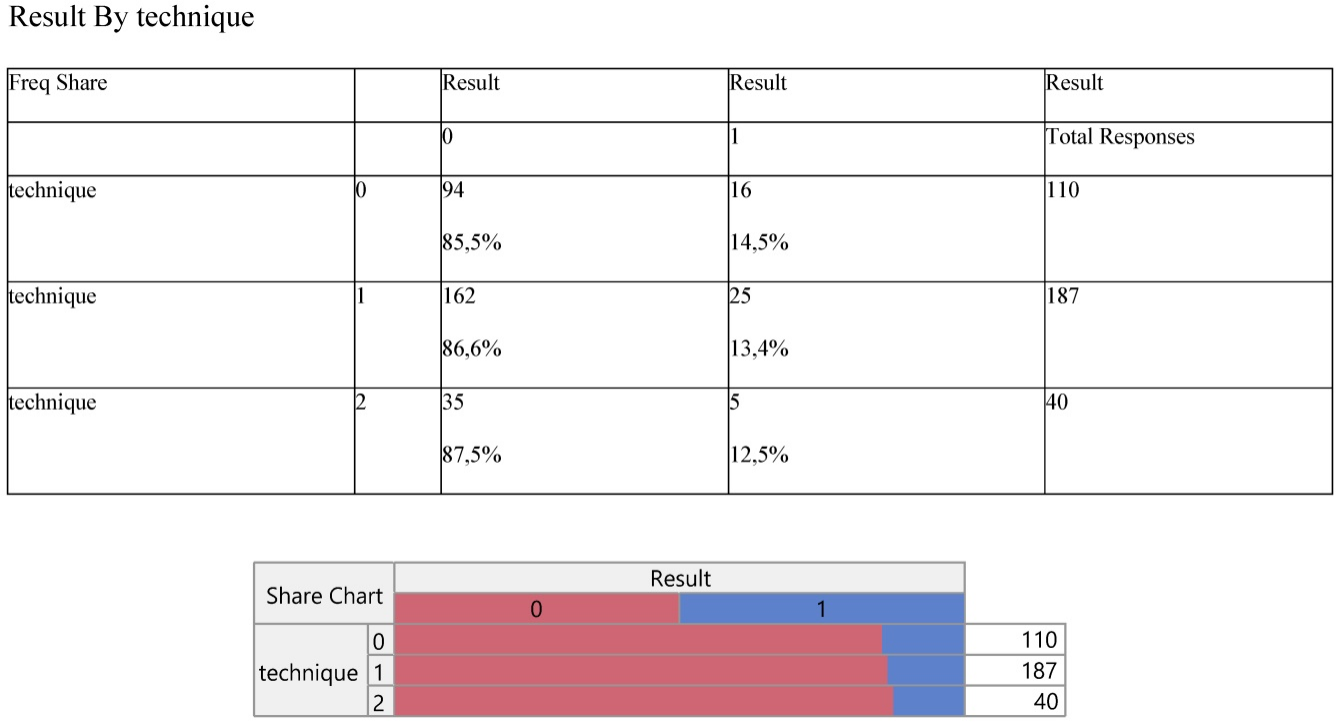
Cross-tabulation between the parameters of the study against the technique. Each cell presents a numerical frequency distribution (freq), percentage contribution across rows (share) and a chi-square p-value. Values less than 0.05 signal for statistical effect. Result; 0=success (pleurodesis), Technique; 0=medical, 1=VATS, 2=conversion

**Table 3:**
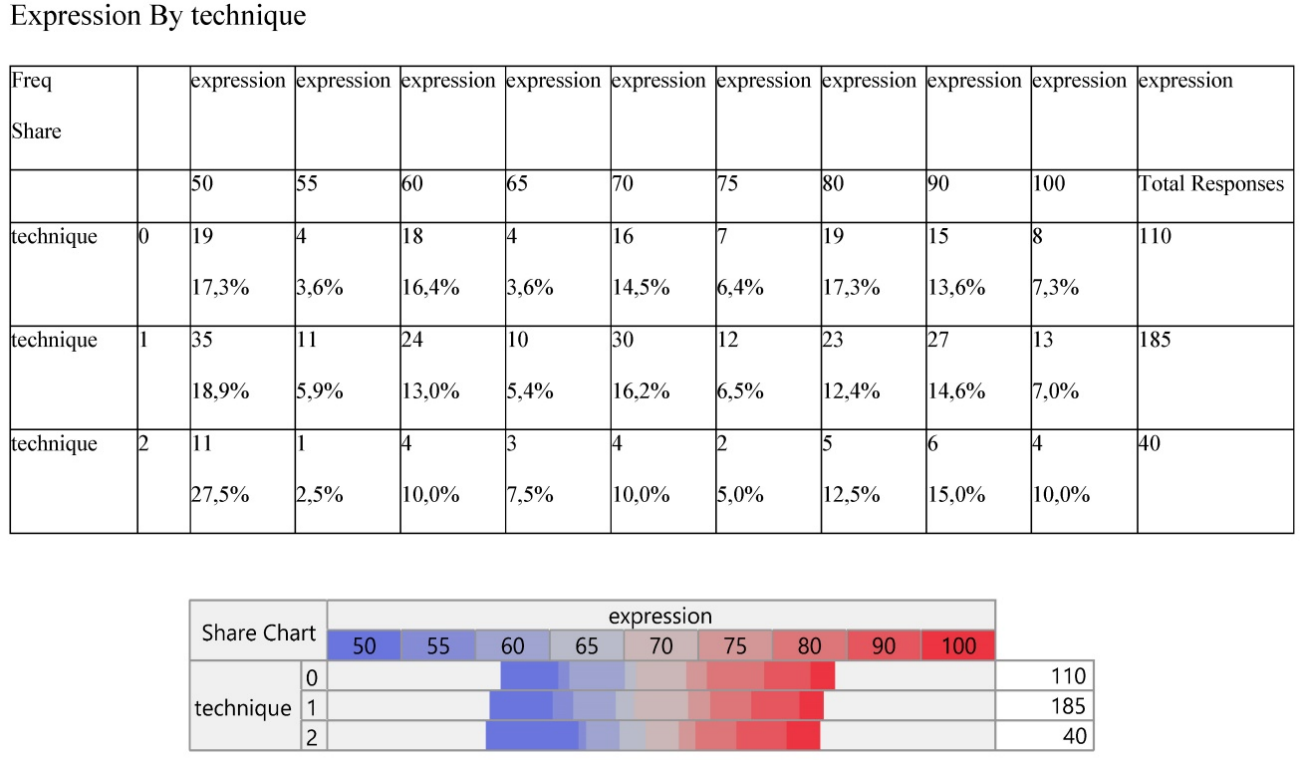
Cross-tabulation between the parameters of the study against the technique. Each cell presents a numerical frequency distribution (freq), percentage contribution across rows (share) and a chi-square p-value. Values less than 0.05 signal for statistical effect. Technique; 0=medical, 1=VATS, 2=conversion, Expression; The level of PD-L1 expression

**Table 4:**
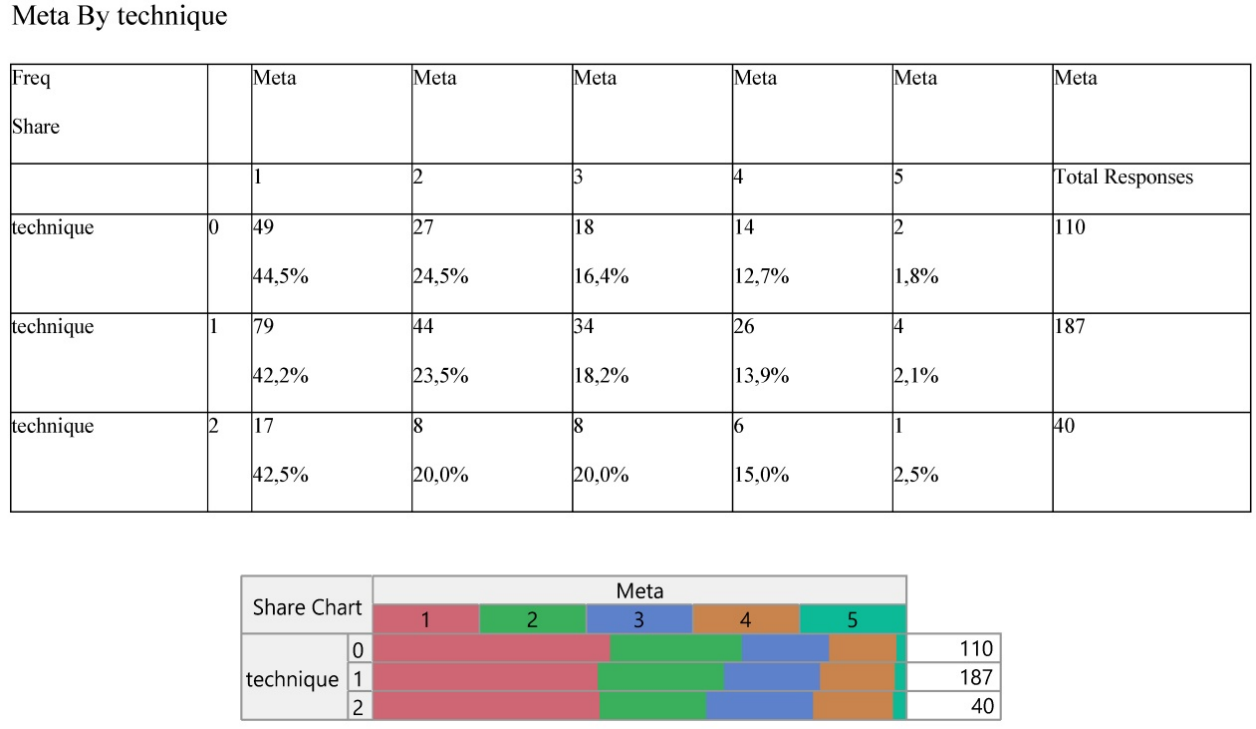
Cross-tabulation between the parameters of the study against the technique. Each cell presents a numerical frequency distribution (freq), percentage contribution across rows (share) and a chi-square p-value. Values less than 0.05 signal for statistical effect. Technique; 0=medical, 1=VATS, 2=conversion, Metastasis; 1=bone, 2=brain, 3=liver, 4=adrenal gland, 5=other, 6=more than 1 metastatic site

**Table 5:**
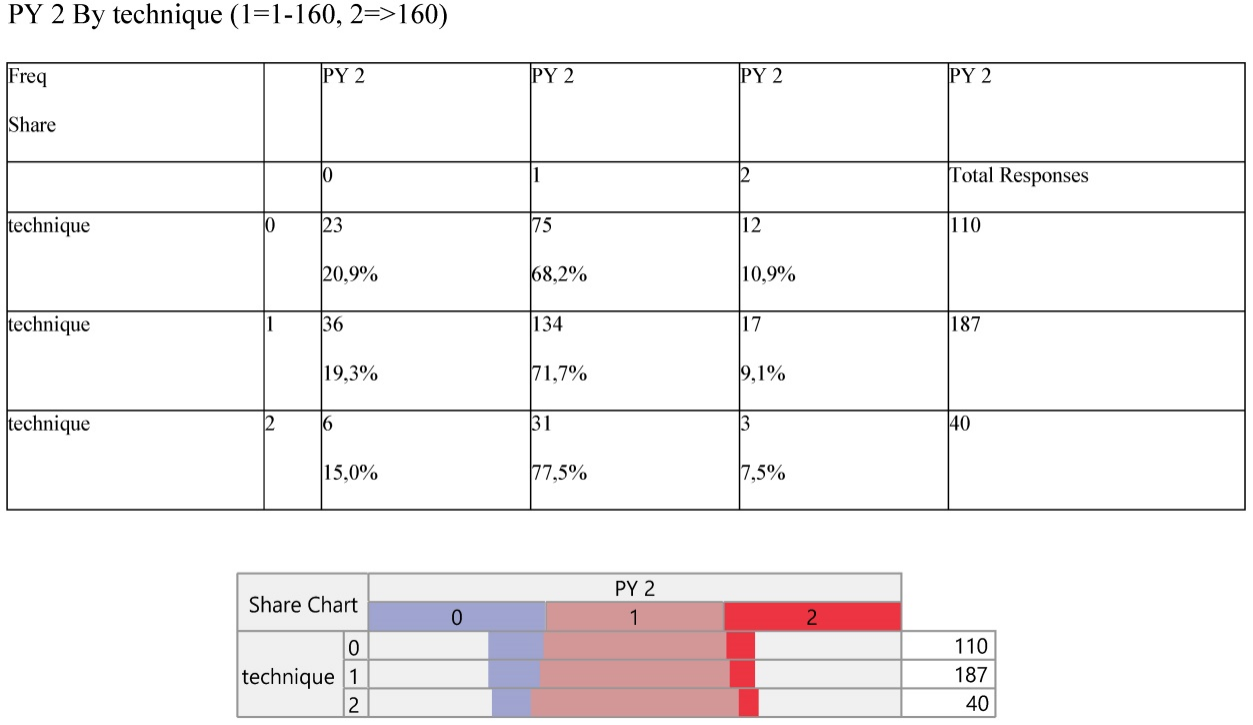
Cross-tabulation between the parameters of the study against the technique. Each cell presents a numerical frequency distribution (freq), percentage contribution across rows (share) and a chi-square p-value. Values less than 0.05 signal for statistical effect. Technique; 0=medical, 1=VATS, 2=conversion,

**Table 6:**
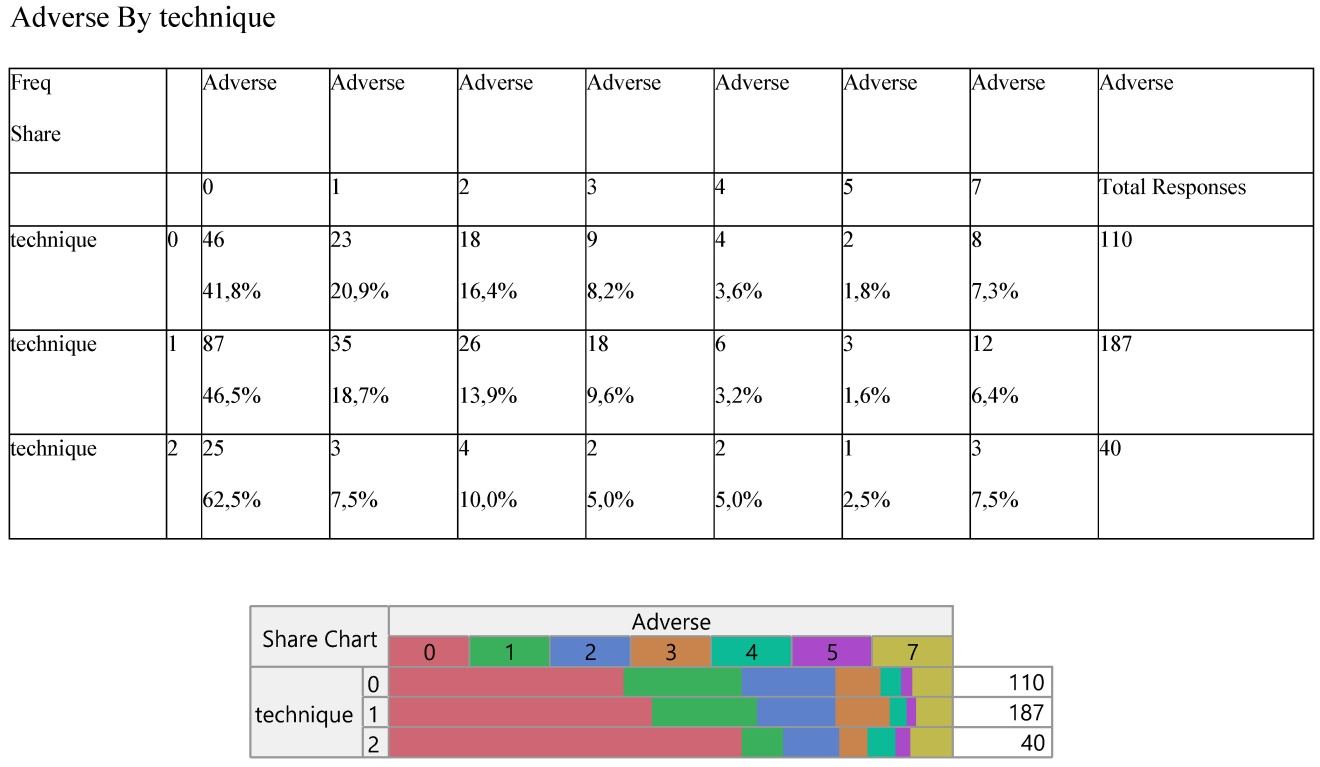
Cross-tabulation between the parameters of the study against the technique. Each cell presents a numerical frequency distribution (freq), percentage contribution across rows (share) and a chi-square p-value. Values less than 0.05 signal for statistical effect. Technique; 0=medical, 1=VATS, 2=conversion, Adverse effects; 0=none, 1=pneumonitis, 2=liver, 3=skin, 4=kidney, 5=thyroid, 6=other, 7=more than 1

**Table 7:**
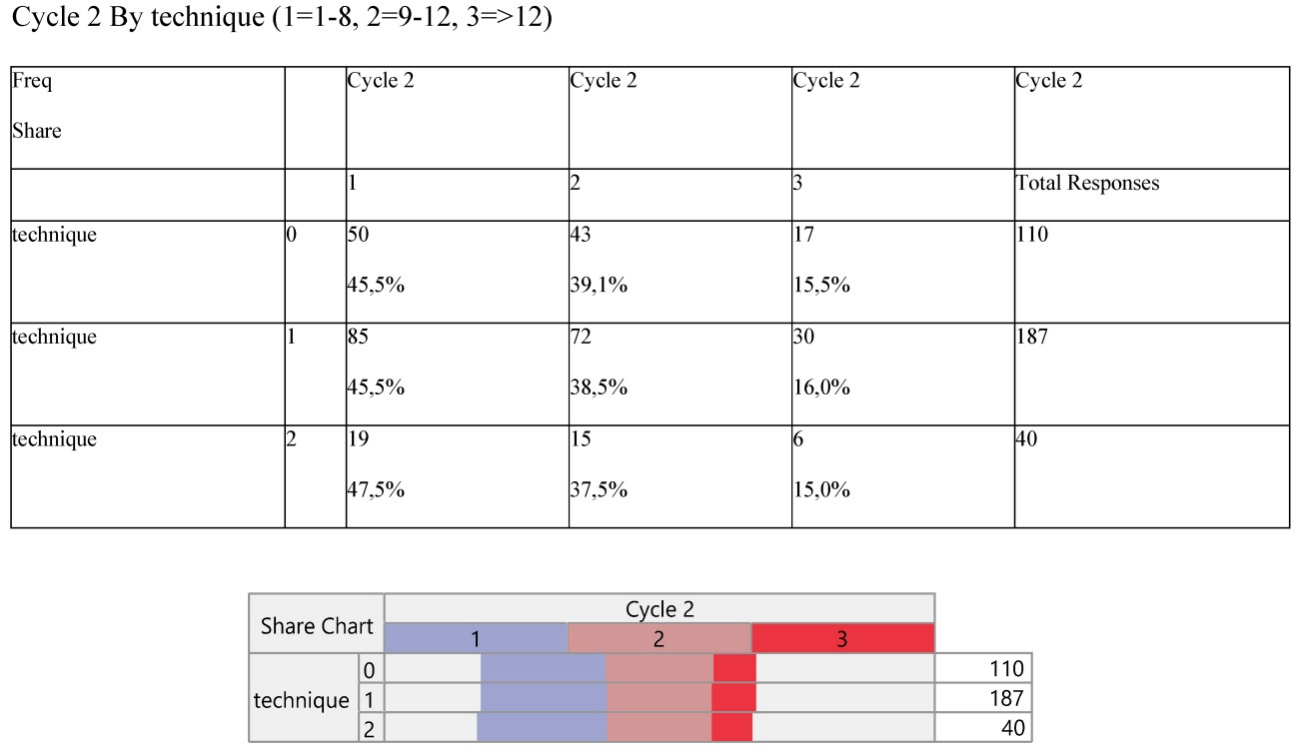
Cross-tabulation between the parameters of the study against the technique. Each cell presents a numerical frequency distribution (freq), percentage contribution across rows (share) and a chi-square p-value. Values less than 0.05 signal for statistical effect. Technique; 0=medical, 1=VATS, 2=conversion

**Table 8:**
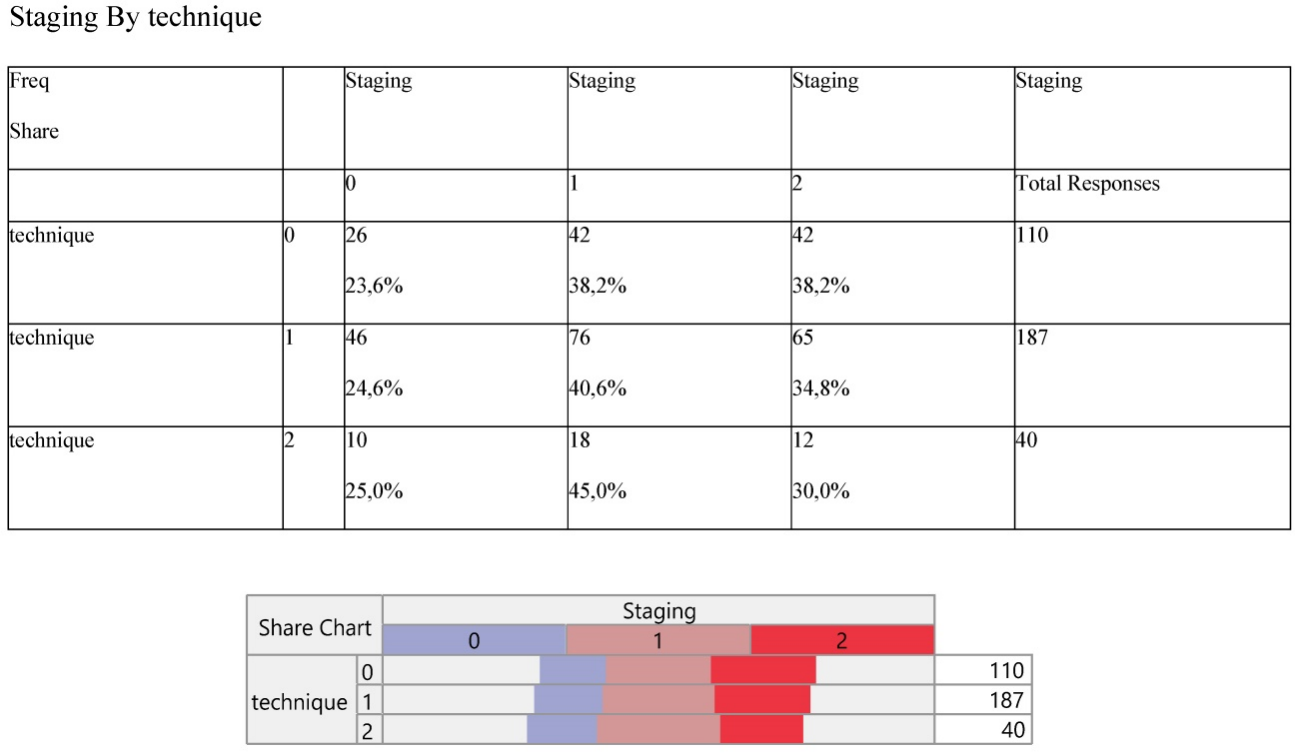
Cross-tabulation between the parameters of the study against the technique. Each cell presents a numerical frequency distribution (freq), percentage contribution across rows (share) and a chi-square p-value. Values less than 0.05 signal for statistical effect. Technique; 0=medical, 1=VATS, 2=conversion, Re-staging upon pleurodesis; 0= Complete Response (CR), 1= Stable Disease (SD), 3= Progressive Disease (PD)

**Table 9:**
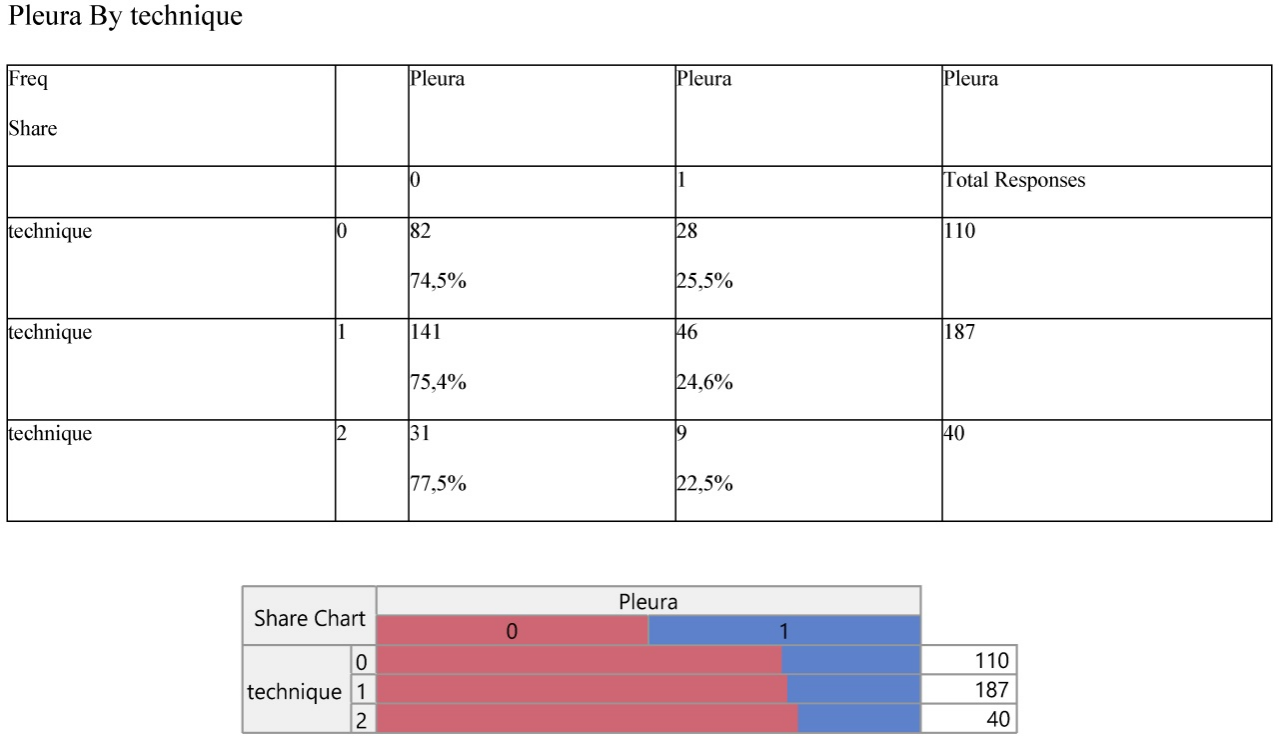
Cross-tabulation between the parameters of the study against the technique. Each cell presents a numerical frequency distribution (freq), percentage contribution across rows (share) and a chi-square p-value. Values less than 0.05 signal for statistical effect. Technique; 0=medical, 1=VATS, 2=conversion, Pleura; 0= pleura effusion upon diagnosis, 1= no pleura effusion upon diagnosis
